# Timing and causes of forest fire at the K–Pg boundary

**DOI:** 10.1038/s41598-022-17292-y

**Published:** 2022-07-29

**Authors:** A. Santa Catharina, B. C. Kneller, J. C. Marques, A. D. McArthur, S. R. S. Cevallos-Ferriz, T. Theurer, I. A. Kane, D. Muirhead

**Affiliations:** 1 School of Geosciences, University of Aberdeen, Aberdeen, AB23 3UE UK; 2 ITT Oceaneon, Universidade do Vale do Rio dos Sinos, Sao Leopoldo, 93020-190 Brazil; 3 Instituto de Geociências, Universidade Federal do Rio Grande do Sul, Porto Alegre, RS 90650-001, Brazil; 4 School of Earth and Environment, University of Leeds, Leeds, LS2 9JT UK; 5 Universidad Autónoma de México, 04510 Ciudad de México, Mexico; 6 Department of Earth and Environmental Sciences, University of Manchester, Manchester, M169PL UK

**Keywords:** Natural hazards, Solid Earth sciences

## Abstract

We report K–Pg-age deposits in Baja California, Mexico, consisting of terrestrial and shallow-marine materials re-sedimented onto the continental slope, including corals, gastropods, bivalves, shocked quartz grains, an andesitic tuff with a SHRIMP U–Pb age (66.12 ± 0.65 Ma) indistinguishable from that of the K–Pg boundary, and charred tree trunks. The overlying mudstones show an iridium anomaly and fungal and fern spores spikes. We interpret these heterogeneous deposits as a direct result of the Chicxulub impact and a mega-tsunami in response to seismically-induced landsliding. The tsunami backwash carried the megaflora offshore in high-density flows, remobilizing shallow-marine fauna and sediment *en route*. Charring of the trees at temperatures up to > 1000 °C took place in the interval between impact and arrival of the tsunami, which on the basis of seismic velocities and historic analogues amounted to only tens of minutes at most. This constrains the timing and causes of fires and the minimum distance from the impact site over which fires may be ignited.

## Introduction

The consensus is that the Chicxulub bolide impact was at least partially responsible for the Cretaceous–Paleogene boundary extinction event, e.g.,^1–3^, and references therein] and associated profound climatic changes, e.g.,^4–6^. Schulte et al.^7^ identified a pattern of decreasing ejecta thicknesses with distance from the impact site consistent with it being the single source, e.g.,^8,9^. This interpretation is also supported by the distribution, composition, and depositional characteristics of the ejecta^1,10–13^.

Continental margin localities proximal to the impact site and on the North Atlantic margin of North America show evidence of large-scale mass wasting, e.g.,^11,14,15^; an iridium anomaly is often observed immediately above the mass flow deposits, associated with other ejecta such as spherules and shocked minerals, e.g.,^7^. There is also evidence of global-scale fires in the form of soot^16,17^, though there has been considerable debate over the exact nature, timing and proximate cause of these fires.

**Figure 1:**
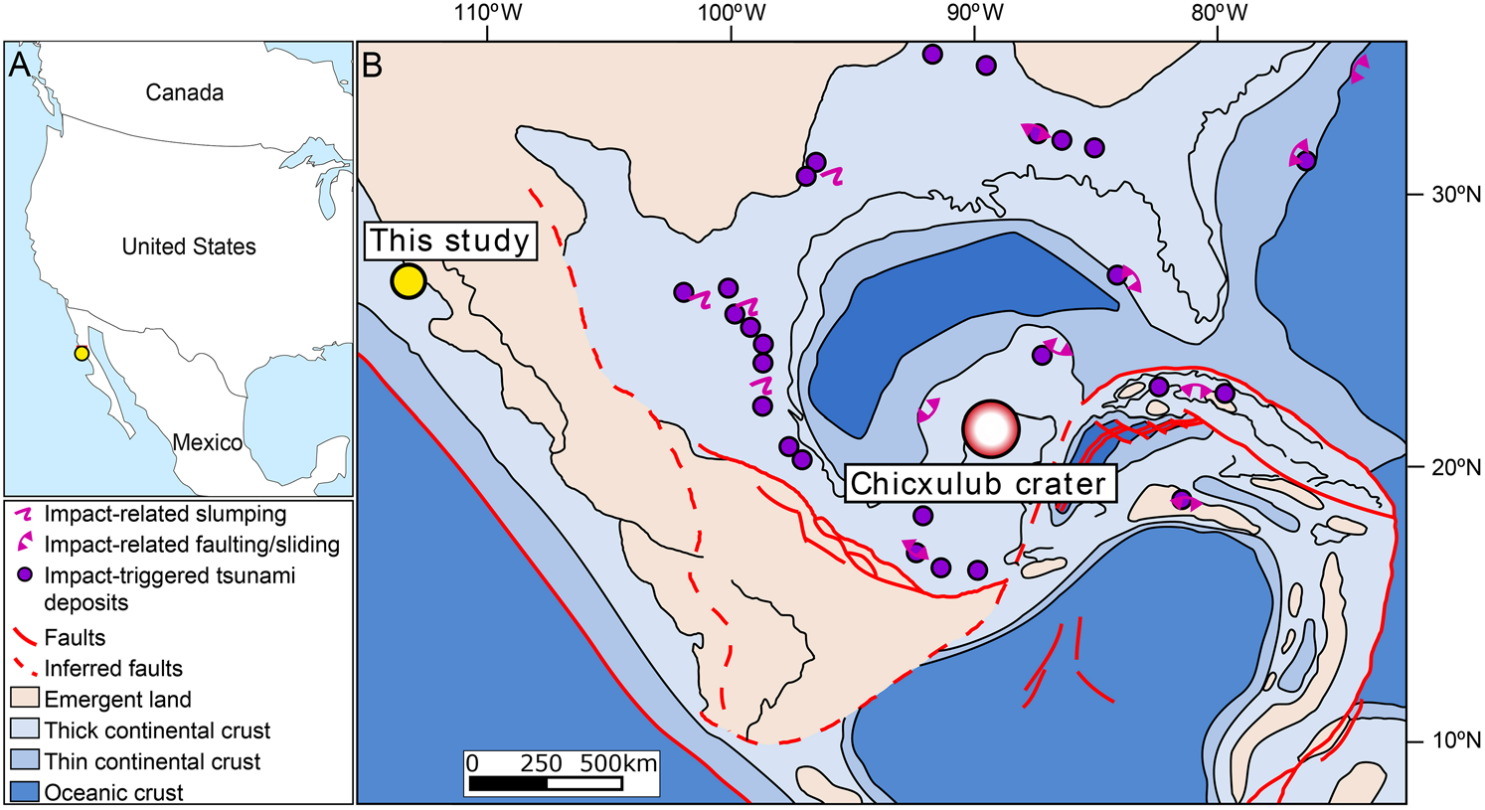
(**A**) location map of the study area (georeferenced and vectorized from Google Earth Pro (2022): https://www.google.com.br/earth/about/versions/), using ArcMap 10.7: https://www.esri.com/en-us/arcgis/products/arcgis-desktop/resources); (**B**) paleogeographic reconstruction of Gulf of Mexico and Baja California Pacific margin taken from Stéphan et al.^18^, and Helenes & Carreño^19^; using ArcMap 10.7: https://www.esri.com/en-us/arcgis/products/arcgis-desktop/resources), with location of this study, Chicxulub crater, and impact-related slumps, faults, slides, and tsunami deposits (compiled by Vellekoop et al.^20^).

**Figure 2:**
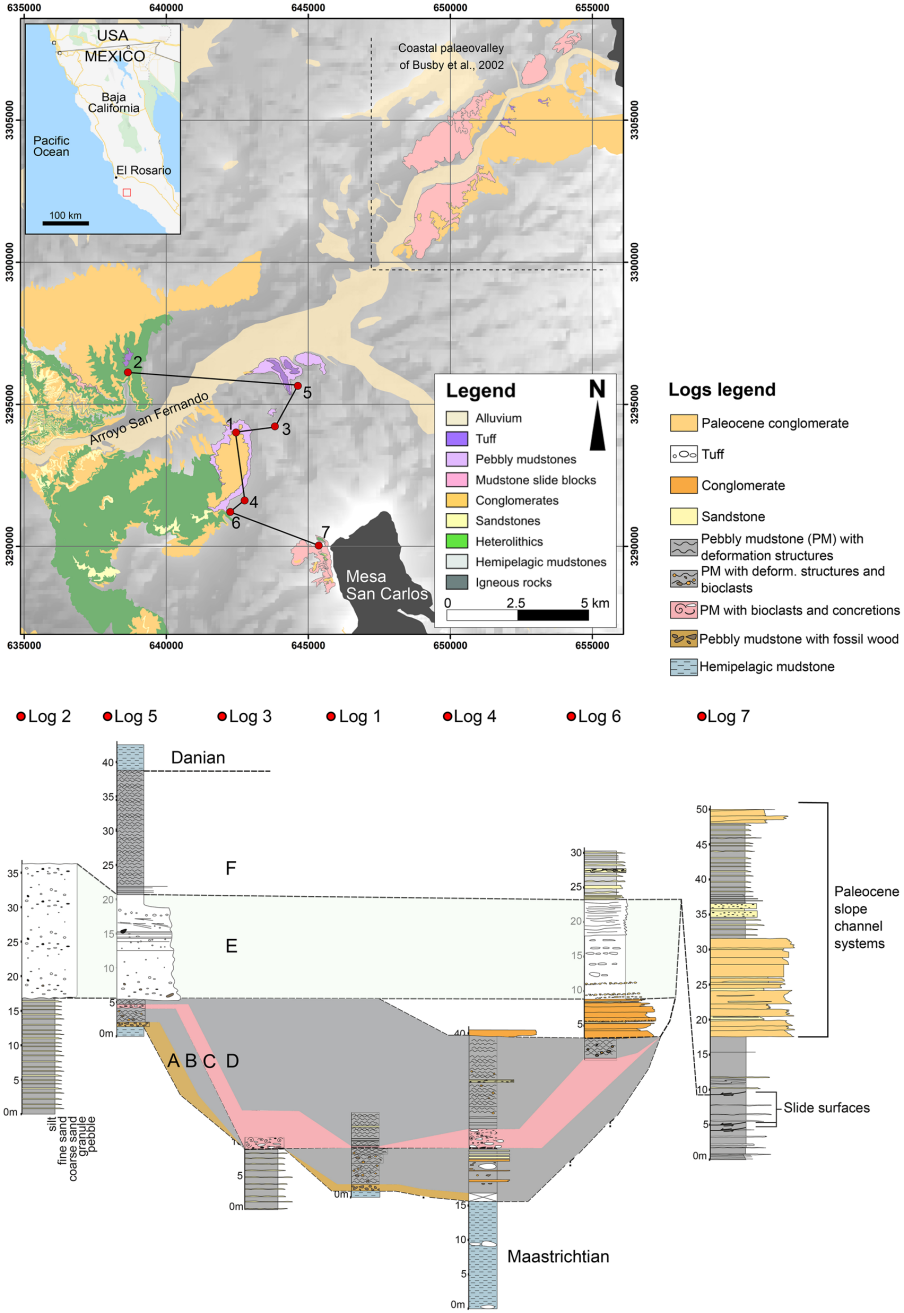
Log correlation of K–Pg-related deposits; main lithologies, structures, and fossils, within upper Maastrichtian and lower Danian hemipelagic mudstones. Panel is hung on correlative horizons that are present in three or more sections. Penecontemporaneous conglomerate deposits are unrelated to the K–Pg event and form part of channel systems that are ubiquitous in this stretch of the Pacific margin. Base map generated from: Esri, HERE, Garmin, Intermap, increment P Corp., GEBCO, USGS, FAO, NPS, NRCAN, GeoBase, IGN, Kadaster NL, Ordnance Survey, Esri Japan, METI, Esri China (Hong Kong), (c) OpenStreetMap contributors, and the GIS User Community. Map produced using ArcMap 10.7: https://www.esri.com/en-us/arcgis/products/arcgis-desktop/resources.

The upper Campanian to lower Danian Rosario Formation near the town of El Rosario on the western side of the Baja California peninsula, c. 300 km south of the US/Mexico border (Fig. 1)^21,22^, comprises c. 1200 m of deep-marine sedimentary rocks^23,24^, and references therein]. It includes several slope channel systems and submarine landslide deposits within background hemipelagic slope mudstones, little faulted and with very low dips, e.g. ^25,26^, (Fig. 2). This west-facing continental margin in the Upper Cretaceous was oriented similarly to the modern coastline, with a shoreline approximately 25 km east of its current location^27^, where shallow marine sediments onlap a Lower Cretaceous arc succession. The study area represents the upper slope, with a slope angle of 3.5 to 7°, estimated from water depth (1500–3000 m based on benthic foraminiferal assemblages^28^); and distance to the contemporaneous shoreline (c. 25 km to NE^27^).

This sequence includes a previously undocumented ≤ 60 m package recording catastrophic re-sedimentation events. The occurrence of terrestrial material (charred tree trunks) and shallow-water micro and macro fauna (corals, gastropods, and bivalves) is in marked contrast to the enclosing bathyal succession^25,26,28^. We first present previously undescribed geological, palaeontological, palynological, and geochemical evidence from this relatively impact-proximal (2500 km) setting to show that this represents the K–Pg boundary succession. It demonstrates the burning of live trees at temperatures consistent with crown fires before the arrival of an impact-related tsunami (the first described from the Pacific margin) that displaced them into deep water on the continental slope. Finally, we discuss the implications for the timing, causes and distance from impact site of wildfires.

## Results

The Maastrichtian/Danian boundary has been mapped in the region as an erosive surface^23^, and wherever the Maastrichtian–Palaeocene boundary can be seen in this area it is truncated by km-scale, low-angle slide surfaces, commonly overlain by slide blocks (Fig. 2). The sedimentary package lying above these surfaces and associated blocks contrasts with the sequences above and below in consisting of pebbly mudstones, locally with shallow-marine foraminifera, horizons rich in terrestrial and shallow-water fossils, and a tuff (Fig. 2). They are immediately succeeded by hemipelagic Danian deposits. No nannofossils or planktic foraminifera were encountered in this package, and benthic foraminifera are decalcified.

### Unit A (pebbly mudstone with fossil wood)

**Figure 3:**
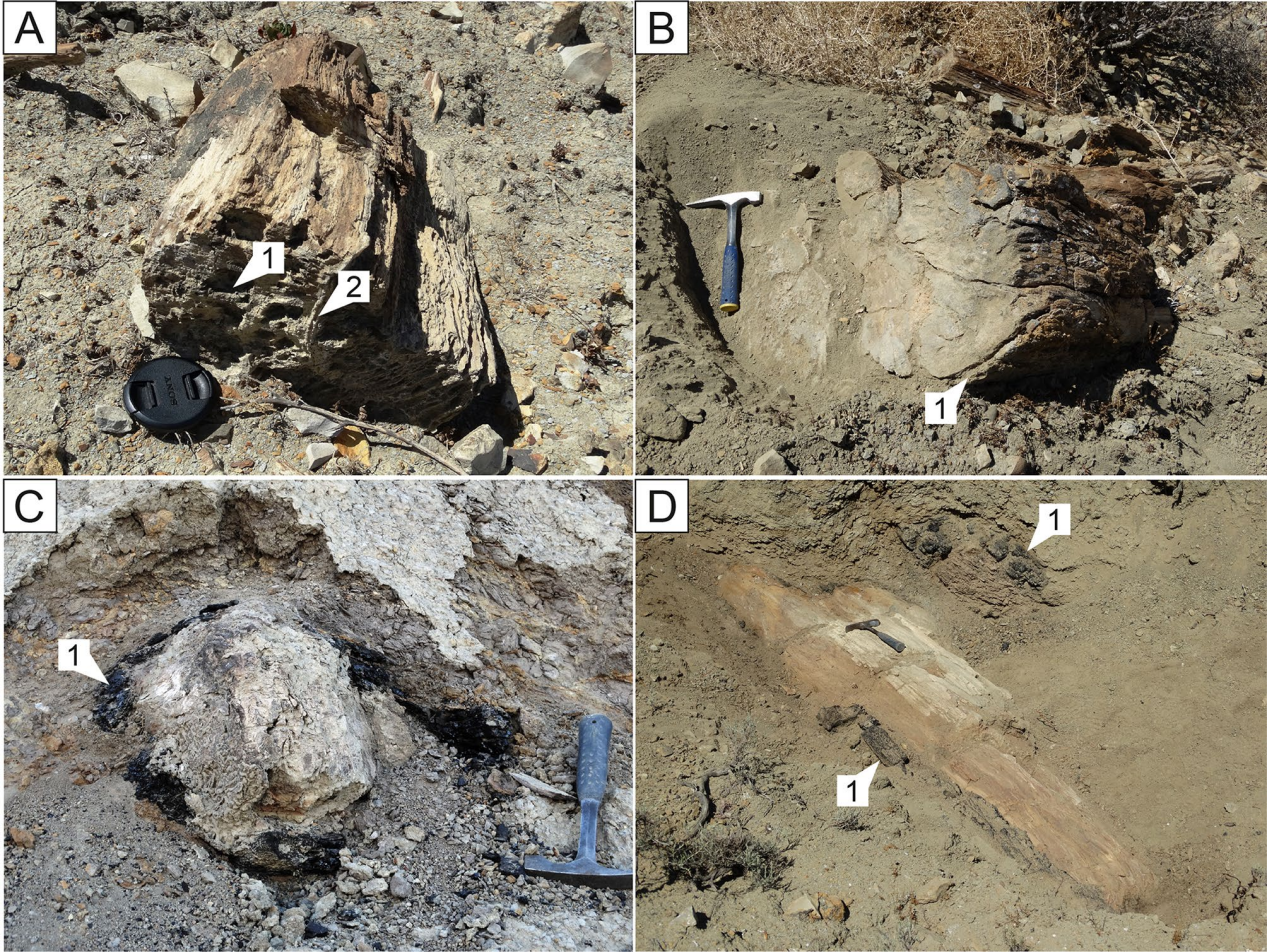
(**A**) Fossil wood with medullary rays (1) and growth rings (2), shown by weathering (lens cap 5 cm); (**B**) Fossil wood with bark preserved indicated by (1) (hammer is 30 cm long); (**C**,**D**) Charred external portions of trunks indicated by (1) (hammer is 30 cm long).

The lowest unit is a ≤ 6 m-thick pebbly mudstone (thinning to both NW and SE; Fig. 2). Charcoalified sub-cm plant fragments comprise 5% of the deposit in a clay-rich matrix. Sparse rounded granules and pebbles of crystalline rock occur throughout. It is distinctive in containing fossilized fragments of tree trunks, including *Pinaceae*, *Cupressaceae* and *Lauraceae* (the new species *Rosarioxylon bajacaliforniensis* Cevallos-Ferriz^29^). The specimens are ≤ 1.5 m in diameter and up to 3 m in length, larger and more common at the base, distributed every few meters horizontally along the outcrop, forming a Lagerstätte. Preservation is exceptional, with nodes and broken branch stumps, bark commonly attached and visible growth rings (Fig. 3A,B). Vascular, epithelial oil/mucilage cells, resin canals and ducts, rays and pits are also well preserved^29^.

Parts of these trunks are charred, with portions of bark and cambium completely charcoalified but still attached to the rest of the trunk (Fig. 3C,D), suggesting very little reworking and rapid burial. The internal wood below the distinct charring layer appears uncharred/unaltered and shows no evidence of decay prior to burning. The absence of bio-erosion, such as *Teredolites*^30^, also suggests negligible post-mortem residence time at air/water and water/sediment interfaces.

**Figure 4:**
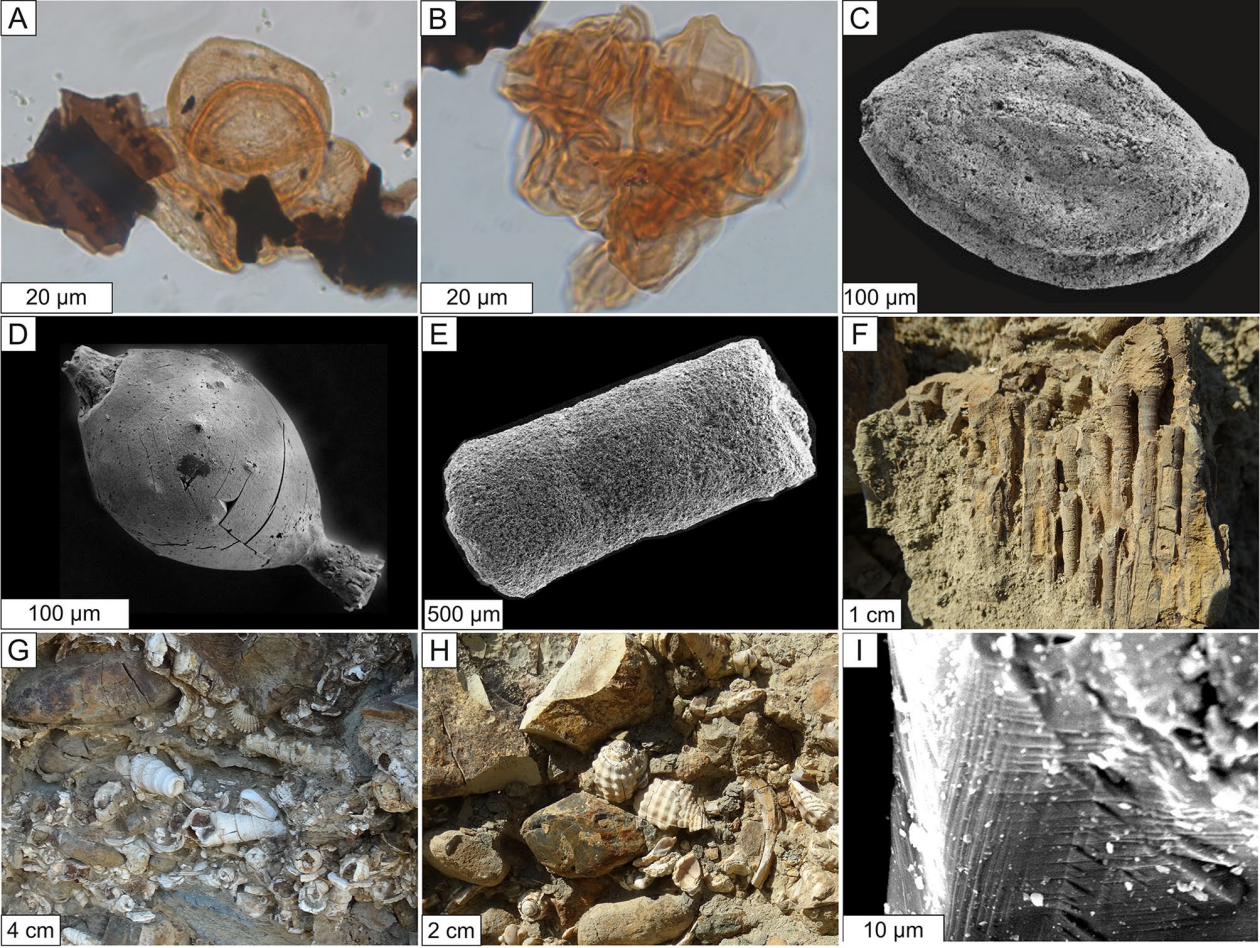
(**A**) *Classopollis* sp. tetrad, slide coordinates C44-4 (sample LOG-01–002); (**B**) Cluster of fungal spores, slide coordinates C43-1 (sample LOG-01 MWD4); (**C**) Miliolid (sample LOG-05–03); (**D**) decalcified Lagenid, (sample LOG-05–21); (**E**) *Bathysiphon* sp. (sample MWD-18); F) *Isopora* sp. corals; (**G**) *Turritella webbi* gastropods, bivalve shells, pebbles, and cobbles; (**H**) *Retipirula crassitesta* gastropods; (**I**) shocked quartz crystal (sample SF-30).

Around 70% of the palynomorphs are dinocysts and acritarchs, including the Cretaceous markers *Dinogymnium* spp. and *Yolkinigymnium* spp. (cf^31^.). The bulk of plant palynomorphs are angiosperm pollens and pteridophyte spores with clear Mesozoic forms (Fig. 4A,B. Spore color indicates burial of < 1 km. No foraminiferal tests were found in this unit (Supplementary Information, Fig. S2 and tables S2-S3).

### Unit B (pebbly mudstone)

This ≤ 8 m thick unit thins towards NW and SE (Fig. 2) and overlaps Unit A, overlying it via a transition over a few cm, containing more silt, less clay (~ 55%) and only sparse sub-dm fragments of plant material. It includes similar granules and pebbles, concretions (≤ 10 cm in diameter), convolute bedding and soft-sediment folds.

Shallow-water benthic foraminifera such as miliolids and lagenids (Fig. 4C,D), cf. Midway Fauna, Gulf of Mexico (GoM)^32^, indicate re-deposition of shelfal and/or upper slope sediments. Unit B yielded few palynomorphs; like Unit A, dinocysts are dominant, but fewer acritarchs were observed. Bryophyte, angiosperm and pteridophyte spores are present.

### Unit C (bioclast-rich pebbly mudstone)

This ≤ 2 m thick, erosively-based unit of silty mudstone contains a high concentration of randomly oriented, 1–5-cm coral fragments (*Flabellum* sp. And *Lithostrotionella* sp.), gastropods (*Turritella* sp., *Retipirula* sp. And *Pyropsis* sp.) and bivalves (*Calva* sp.) throughout (e.g., Fig. 4F–H). Preservation is excellent, with little abrasion (few fragmented edges, shell ornamentation intact), and bivalves frequently found still articulated with closed valves, suggesting transport with minimal turbulence in a cohesive flow. Reworked fossil wood fragments occur throughout, along with concretions, both ranging from a few centimeters to 1 m. Palynomorph recovery and preservation were poor in this unit.

### Unit D (pebbly mudstone)

This ≤ 15 m thick unit has a sharp, flat contact, and is indistinguishable from Unit B, apart from its lower organic content. The palynomorph assemblage, however, comprises more than 80% fungal spores (Fig. 4B) that indicate the presence of a terrigenous component, possibly remobilized soil.

### Unit E (tuff)

This ≤ 20 m thick unit of lapilli crystal vitric tuff has an erosive base, and its lower half incorporates crystalline pebbles and fossil tree trunks from the underlying units. Its margins are not seen, but it thins overall towards both NW and SE. Busby et al.^27^ described the proximal equivalent to this unit c. 25 km to the NE. The tuff is of intermediate composition (70% vitreous groundmass, 15% labradorite, 5% quartz, 6% biotite, 3% hornblende, with subordinate lithic fragments, oxides, and zircon), and includes rare, shocked quartz ≤ 150 µm (Fig. 4I). It fines upwards overall, with bands of coarse ash alternating with pumice lapilli (≤ 5 cm diameter in this section and bombs ca. 35 cm in the area 25 km to the NE). Our SHRIMP U–Pb dating of zircons yields an age of 66.12 ± 0.65 Ma (Supplementary Information, Fig. S2 and Table S4). Within error, the tuff age we obtained is indistinguishable from the 65.5 ± 0.6 Ma Ar–Ar biotite age of the proximal tuff^27^, both coeval with that proposed for the Chicxulub impact, 66 Ma^33^.

### Unit F (pebbly mudstone)

The ≤ 18 m thick unit above the tuff (Fig. 2) is similar in texture and composition to Units B and D, but its largest clasts rarely exceed 2 cm in diameter. Poor exposure and surface alteration obscure any deformation structures. No palynomorphs were recovered from this unit; the few benthic foraminifera recovered were etched and fragmented, implying reworking.

### Danian mudstones

**Figure 5:**
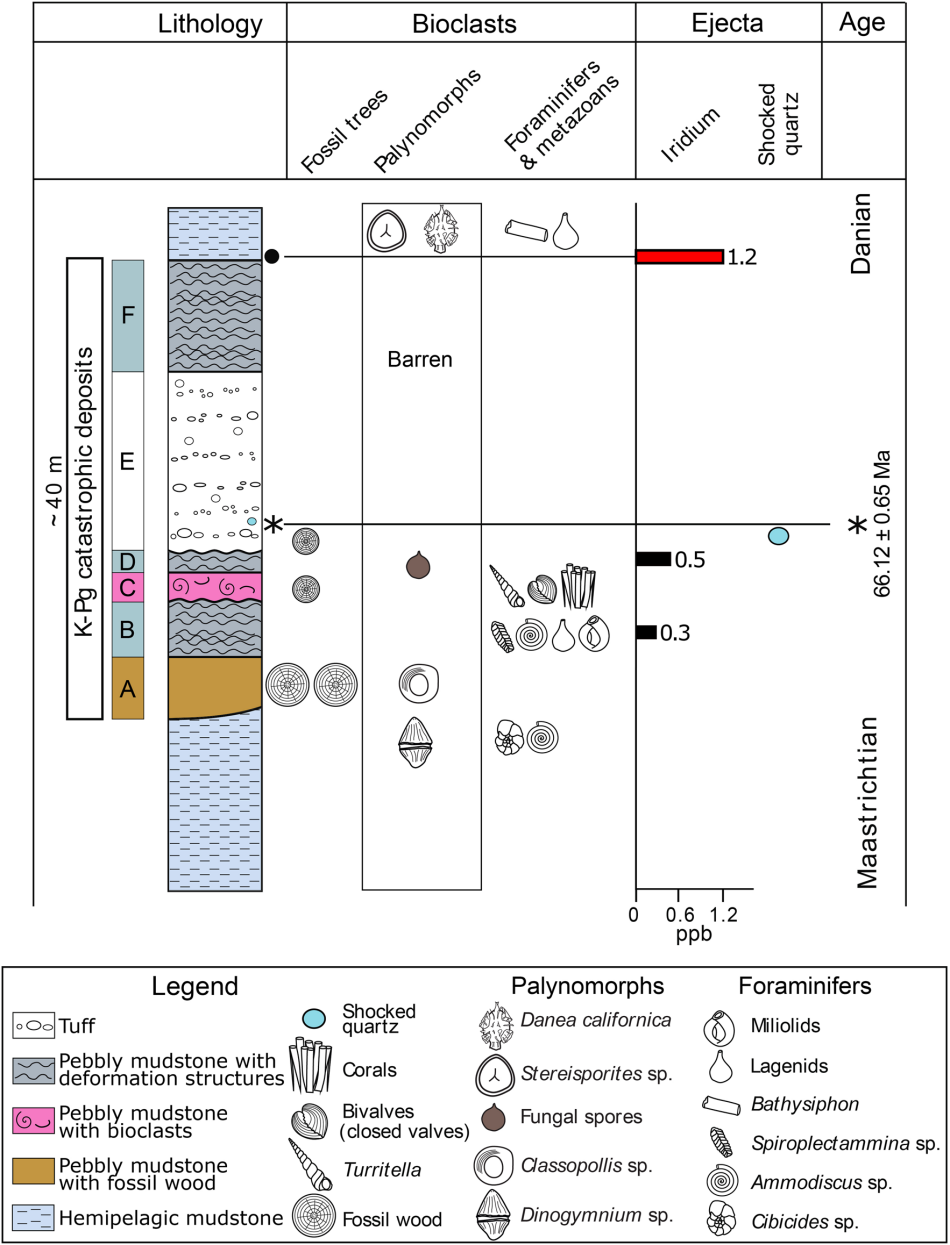
Generalized section of K–Pg catastrophic deposits in this study, showing lithologic units, the occurrence of fossilized tree trunks, occurrences of selected abundant palynomorphs, foraminifers and metazoan taxa, iridium concentrations, shocked quartz crystal occurrence, and absolute age.

The mudstones immediately above Unit F contain bathyal benthic foraminifera (mostly *Bathysiphon* sp., Fig. 4E, and lagenids) and an iridium anomaly (1.2 ppb Ir, cf. Units B—0.3 ppb, and D—0.5 ppb; Fig. 5) comparable to K–Pg boundary interval deposits elsewhere, e.g., Brazos River^34,35^, where in proximal and intermediate settings the iridium anomaly often occurs atop mass flow clastic units, e.g.,^7^.

Pteridophytes spores account for 17% of a palynomorph assemblage dominated by fungal spores (Fig. 5; Supplementary Information, Fig. S2) as well as Danian dinocysts (e.g., *Damassadinium californicum*). High concentrations of horizontally disposed *Bathysiphon* sp. also occur, and bioturbation is intense, with sub-centimetric sand-filled burrows constituting up to 40% of these mudstones.

## Discussion and conclusions

The tuff age we obtained is identical within error to that proposed for the Chicxulub impact and the K–Pg Boundary. The apparent absence of spherules and tektites is to be expected given the mixing and dilution within the large volume debris flows and subsequent alteration to clay minerals similar to the muddy debrite matrix. The stratigraphic position, architectural characteristics, radiometric ages, shocked quartz, iridium anomaly, and fungal and fern spore spike all indicate that this was coincident with the K–Pg event (Fig. 5).

The occurrence of fossil trees in Unit A, shelfal macrofauna in Unit C, and shallow-water benthic microfauna throughout, clearly indicate downslope remobilization. Pebbly mudstones such as those in units B, D and F are common in deep marine settings and are generally regarded as deposits of debris flows; the overall similarity of these units suggests a single composite event consisting of more or less continuous debris flow punctuated by faster and more turbulent surges represented by units C and E. Occurrence of exclusively unbioturbated wood within Unit A unequivocally shows the transport of material from a subaerial setting. Transport of wood (presumably buoyant) into the deep marine environment requires buoyancy less than the yield strength of the debrite matrix (cf^36^. and discussion thereof).

Mass failures at many localities around the GoM and the Atlantic margin are attributed to impact-related seismicity, of likely magnitude M10-11 (e.g.^37^,). We interpret the observed large-scale and widespread sliding in the local coastal and slope settings of the Rosario Formation similarly (this study and^27^). Based on the mappable extent of the mass failures, we estimate a minimum volume of 15 km^3^ in the immediate area alone.

Impact-related tsunamis have been invoked for the onshore-directed transport of material in the US Western Interior^37^, and for offshore-directed transport by tsunami backwash around the GoM^8,36^. Numerical modelling of the impact-generated tsunami in the GoM, constrained by tsunami-related deposits on the Atlantic margin, GoM and Caribbean, e.g.,^14^, appears to rule out propagation of significant tsunami waves through the still-open passage from the Caribbean to the Pacific^38^. We therefore attribute tsunami generation to the observed massive seismogenic slope failure on the Pacific margin.

With P wave velocities of 6 to 8 km s^−1^, e.g.,^39^, depending on the ray path, arrival times would have been between 5 and 7 min post-impact. Based on historic seismogenic mass failures, e.g.,^40^, sliding would have occurred almost immediately, and the associated tsunami would have arrived within minutes to tens of minutes, by which time the trees were already charred. The picture is thus one of seismically-triggered landsliding and large-scale re-sedimentation of terrestrial and shallow-marine material by sediment gravity flows. The tsunami (the first of this age recorded from the Pacific margin) is likely to have propagated across the paleo-Pacific to affect many coastal regions of the Earth within days of the impact.

The global presence of soot in K–Pg sequences has been used to argue either for wildfires or for combustion of mineral sources in sedimentary rocks and fossil hydrocarbons close to the impact site, e.g.,^41^, and references therein]. However, uncertainties remain over the total mass of soot, the abundance of burn-related charcoal^42,43^, the heat flux required to ignite live or dead vegetation, and the area over which the requisite heat flux could have occurred, e.g.,^44^. Also uncertain is the timing of the fires, either from the impact plume, e.g.,^45,46^, from ejecta re-entering the atmosphere, e.g.,^16,47,48^, both of which are subject to uncertainties in impact energy, angle, and trajectory, or due to subsequent lightning strikes over the following days to months in forest dead or dying from the after-effects of the impact^23^.

The presence of charcoal fragments and partially-charred fossilized tree trunks in the Rosario Formation demonstrates that the adjacent coastal vegetation suffered some high-intensity thermal event sufficient to cause combustion. The absence of any signs of decay implies that the trees were live when ignited and that charring of the trees was specifically a result of the impact event. Material that was charred prior to the event is likely to have shown microbial degradation in the wood structure, so we can be confident that the charring was a consequence of this event and not a pre-impact wildfire. Charcoalified parts still attached to trunks indicate little exposure to the air–water or water–sediment interface, since charcoal is extremely physically mobile and erodible^49^. Raman spectroscopy yields temperatures between 395 and 1022 °C, with a median of 716 °C.

This high-temperature event occurred before the tsunami, i.e., within minutes to tens of minutes after impact; the timing is thus consistent with ignition either by thermal radiation emitted from the plume^46^ or by ejecta re-entry^48^. The context and nature of preservation of charred material at this site suggest that wildfires began almost immediately after impact and may have begun more easily than suggested by Belcher et al.^44^. The discrete charcoal layer on the tree trunks would be consistent with ‘flash-heating’ alone (e.g., radiation) or of the consequent wildfire that was rapidly quenched by the tsunami, and therefore unable to alter significantly the wood below the outer surface charcoals. However, wildfire propagation would have to have been extremely rapid in the latter case. Further work would be required to differentiate with certainty between these two possibilities.

## Methods

Samples for all analyses presented in this paper were collected along Log 5, at the positions indicated on Supplementary Figure S1, aimed at obtaining a complete profile through the local K–Pg boundary section, with palynomorphs, foraminifera, macro charcoal, fossil wood, materials for dating, and iridium content, to ascertain their correct relative stratigraphic position, and to correlate with other K–Pg sections globally. All figures and tables related to the methods applied are available as Supplementary Information.

### Palynology

Seventeen samples were collected through Log 5 (Supplementary Fig. S1) and were prepared at the Marleni Marques Toigo Laboratory of Palynology at UFRGS, Porto Alegre, Brazil, using the methodology in Supplementary Table S1. Counts of two hundred palynomorphs per slide were made where possible. Biostratigraphic determinations were made utilizing first occurrence, last occurrence, and acme zones previously defined onshore and offshore Mexico^31,50^ and the USA^51–55^ to help determine the relative age. In addition, the color of abundant spore *Stereisporites* spp. was recorded using the standard spore color index methodology^56^.

The lowermost samples, collected from a blue-gray mudstone (Unit A) demonstrate a diverse assemblage of Maastrichtian palynomorphs, including pollen (e.g., *Classopollis* spp., *Tricolporopollenites* spp.), spores (e.g., *Biretisporites* spp. and *Todisporites* spp.), and dinoflagellate cysts, including the Cretaceous markers *Dinogymnium* spp. and *Yolkinigymnium* spp., with representatives of fifty-four genera of both marine and terrestrial forms (Supplementary Fig. S2).

Above the bioclastic debrite, the abundance and diversity are severely reduced, with only seven genera and the sample dominated by fungal spores (Fig. S2). 18 m of section above the lapilli tuff is barren of palynomorphs, but rich in degraded humic debris. At the top of the study interval, a spike in fern spores is observed (e.g., *Baculatisporites comaumensis* and *Laevigatosporites* spp.), along with diminutive Danian dinocysts (e.g., *Damassadinium californicum*) and a distinct absence of any Cretaceous palynomorphs. The count data are presented in Supplementary Table S2, where general groups of palynomorphs are also presented to demonstrate the wider variation in floral abundance.

### Foraminifers and macro charcoal quantification

Separation of foraminifera tests and macro charcoal fragments was carried out concomitantly. Fifteen samples were collected through Log 5 (collocated with samples for palynology, as shown in Fig. S1). Preparation and identification were conducted following standard procedures, e.g.,^57^, but the friable nature of the material simplified and shortened the process: 200 g fractions (and in some cases 400 g, for samples where recovery was poor) of each sample were immersed in a container with distilled water. After a few minutes, the samples were gently disaggregated manually and filtered in a 63 μm sieve to remove the clay minerals. The remaining material was then dried in an oven at a controlled temperature of 40° Celsius.

Selection of specimens was conducted using a Leica S6D stereoscopic microscope, and identification of the foraminifers was conducted using the ACEMAC Nano Scale Electron Microscopy and Analysis Facility at the University of Aberdeen, with the Carl Zeiss Gemini SEM 300—high resolution Field Emission Scanning Electron Microscope (FESEM). Two hundred foraminifera specimens were counted and identified for each sample when possible, and the data are presented on Table S3. Macro charcoal particles were counted, and their total volumetric estimates were established based on the original volume of each sample.

### Fossil wood

The material consists of three trunks collected from Log 5 site (Supplementary Fig. S1, location map in Fig. 2) that represent monopodial trees preserved as silica permineralization. Due to the size of these trunks, the sampling method implemented consisted in removing a fragment of its outermost wood and collecting dispersed wood fragments. Subsequently, the samples were transferred to the Paleobotany Laboratory of the Institute of Geology, UNAM, where they were cut to obtain sections in the three cutting planes (transverse, radial and tangential) used for wood anatomical studies. Conventional thin section techniques were applied. The photomicrographs were obtained with a Canon PowerShot A640 camera and a Carl Zeiss AxioCam ICc 5. Subsequently, they were assembled into photographic plates aided with Illustrator CS4 program.

The recognition of the anatomical characters was based on^58–62^. For quantification, 20 measurements were obtained per attribute, and for ray height, 35 measurements were made. Subsequently, for each characteristic, its average minimum and maximum values were obtained, expressed as follows: average (minimum–maximum) unit. Regarding the measurement and quantification of the tracheid radial pitting, the contiguity index (Cp) and seriation index (Si) of^63^ were followed.

For the taxonomic identification of fossil woods at genus level, we followed^61,62,64–66^. For species level identification, comparisons were made with conifer wood of extant species based mainly on^59,67,68^; while comparisons with fossil wood articles describing wood of the same or similar taxa were also used, e.g.,^69–73^.

### Raman spectrometry

Raman spectra were obtained through random sampling of individual charcoal fragments (n = 50) with no additional treatment. Charcoal sampling surfaces were selected for high reflectivity where possible to ensure an adequate spectral response. A laser power of < 0.3mW was applied over 3 accumulations, totalling 15 s exposure per sample. No combustion damage was observed on laser-irradiated surfaces post-exposure. All spectra were deconvolved within Renishaw WiRE 3.4 software, applying smoothing and a cubic spline interpolative baseline, and bands D and G fit solely. For geothermometric purposes, parameter FWHMRa (D- and G-band width ratio) was utilised within the following equation^74^

$$Formation \;temperature \;( \circ {\text{C}}) = \frac{{\left[ {FWHMRa} \right] - 3.1765}}{ - 0.0016}$$

See also Supplementary Information, Table S4, Fig. S3. Statistical analyses were conducted in IBM SPSS v. 25 via histogram and boxplot presentation (Supplementary Figures S4 and S5, Table S5).

### SHRIMP U–Pb zircon dating

For SHRIMP U–Pb zircon analyses, 1.5 kg of a rock sample of the tuff (SF-30, indicated in Fig. S1) were crushed, powdered, and sieved. Heavy mineral concentrates were obtained by panning, and purified using heavy liquid procedures. Grains were set in epoxy resin mount (together with the Temora zircon standard) and polished. Backscattered electron and cathodoluminescence images were obtained for better spot targeting using a FEI-QUANTA 250 scanning electron microscope equipped with secondary-electron and cathodoluminescence (CL) detectors. The analyses were performed in a SHRIMP (sensitive high-resolution ion microprobe) IIe/MC at the Center of Geochronological Research of the University of Sao Paulo (CPGeo-USP) following the procedures described by^75^. ^206^Pb/^238^U ratio was calibrated using the standard Temora^76^. Measured ^204^Pb was applied for the common lead correction^77^.

Data reduction, plots and calculated ages were carried out using Excel spreadsheets with the support of Squid 2.0^78^ and Isoplot 3.0^79^. A more detailed description can be found in^80^. Twenty-eight grains were analyzed, and the data are presented in Supplementary Table S6. Supplementary Figure S6-A shows all results in the concordia diagram; three analyses were interpreted as from detrital or inherited older grains (in green), and one analysis was considered an outlier (in blue). The 24 remaining analyses yielded a 66.12 ± 0.65 Ma concordia age (2 sigma error) (Supplementary Fig. S3-B) and the same result was obtained from ^206^Pb/^238^U weighted mean age (2 sigma error) (Supplementary Fig. S3-C).

### Iridium analysis

Three samples were collected for iridium content analysis by NiS Fire Assay-Instrumental Neutron Activation Analysis (INAA) (indicated in Supplementary Fig. S1): two (Logs 5–7 and Logs 5–21) from the pebbly mudstones of the K–Pg deposits and one (MWD-18) of the hemipelagic mudstone immediately above. They were pulverized to a nominal 2 mm, mechanically split to obtain a representative sample and then pulverized to at least 95% passing—105 μm or smaller. Samples were subsequently transferred to the ACTLAB facilities in Ontario, Canada, and analyzed following the procedures described in^81,82^.

25 g of each sample, along with 2 blanks, 3 certified standards and 3 duplicates, were fire assayed using nickel sulfide (NiS) fire assay procedure. The nickel sulfide button was then dissolved in concentrated HCl, and the residues of this reaction, containing all the iridium (and other PGE) were then collected on a filter paper. This residue was then submitted to two irradiations and three separate counts to measure all the elements.

Iridium concentrations for the three samples analyzed and used in this paper are presented in Table S7, along with detection limits of the method used.

## Supplementary Information

Supplementary Information.

## Data Availability

All data generated or analyzed during this study are included in this published article (and its supplementary information files). Samples collected and prepared for all laboratory analyzes are available upon request.
