# Supplementary material for: Timing and causes of forest fire at the K–Pg boundary
Source: Sci Rep. 2022 Jul 29;12:13006. doi: 10.1038/s41598-022-17292-y (PMC9338043; doi:10.1038/s41598-022-17292-y)
Supplement: Supplementary file 1 — Supplementary Information. [file 41598_2022_17292_MOESM1_ESM.docx]

**SUPPLEMENTARY INFORMATION**


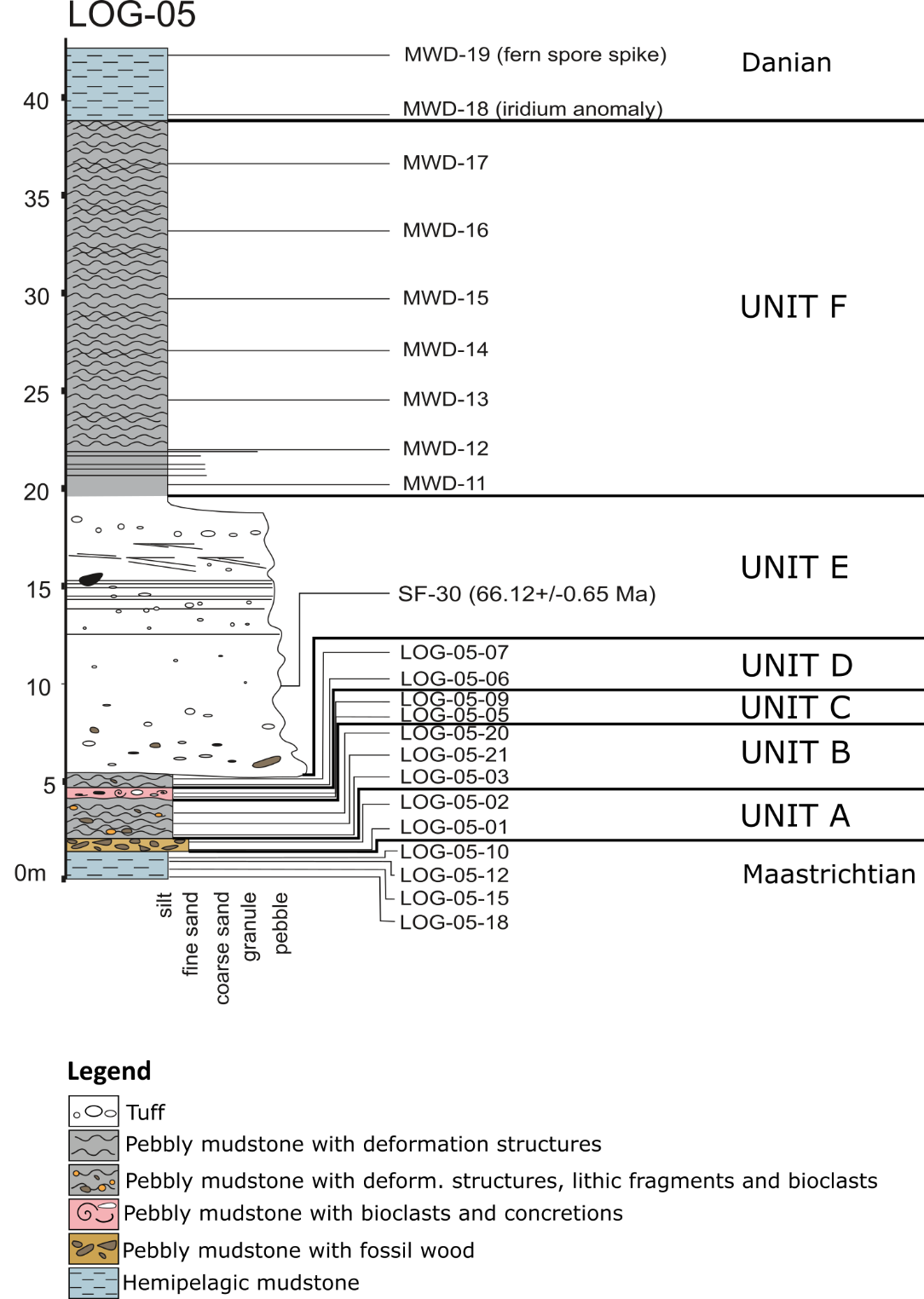


**Figure S1. Log of the K-Pg section, showing lithologies, sample locations, tuff age, iridium anomaly and fern spore spike. Units A-F indicated correspond to units described in the manuscript.**

**Table S1. Palynological slide preparation methods.**

| 10 g samples washed, disaggregated to fragments 1 cm^3^ in size |
| --- |
| 37% cold HCl for 2 hours, dilute to neutral with distilled water |
| 48% cold HF for 24 hours, dilute to neutral with distilled water |
| 37% cold HCl for 1 hour, dilute to neutral with distilled water |
| 10% cold KOH for 10 minutes, dilute to neutral with distilled water |
| 10 μm sieve, palynofacies slide mounted with cellosize and entellan glue |


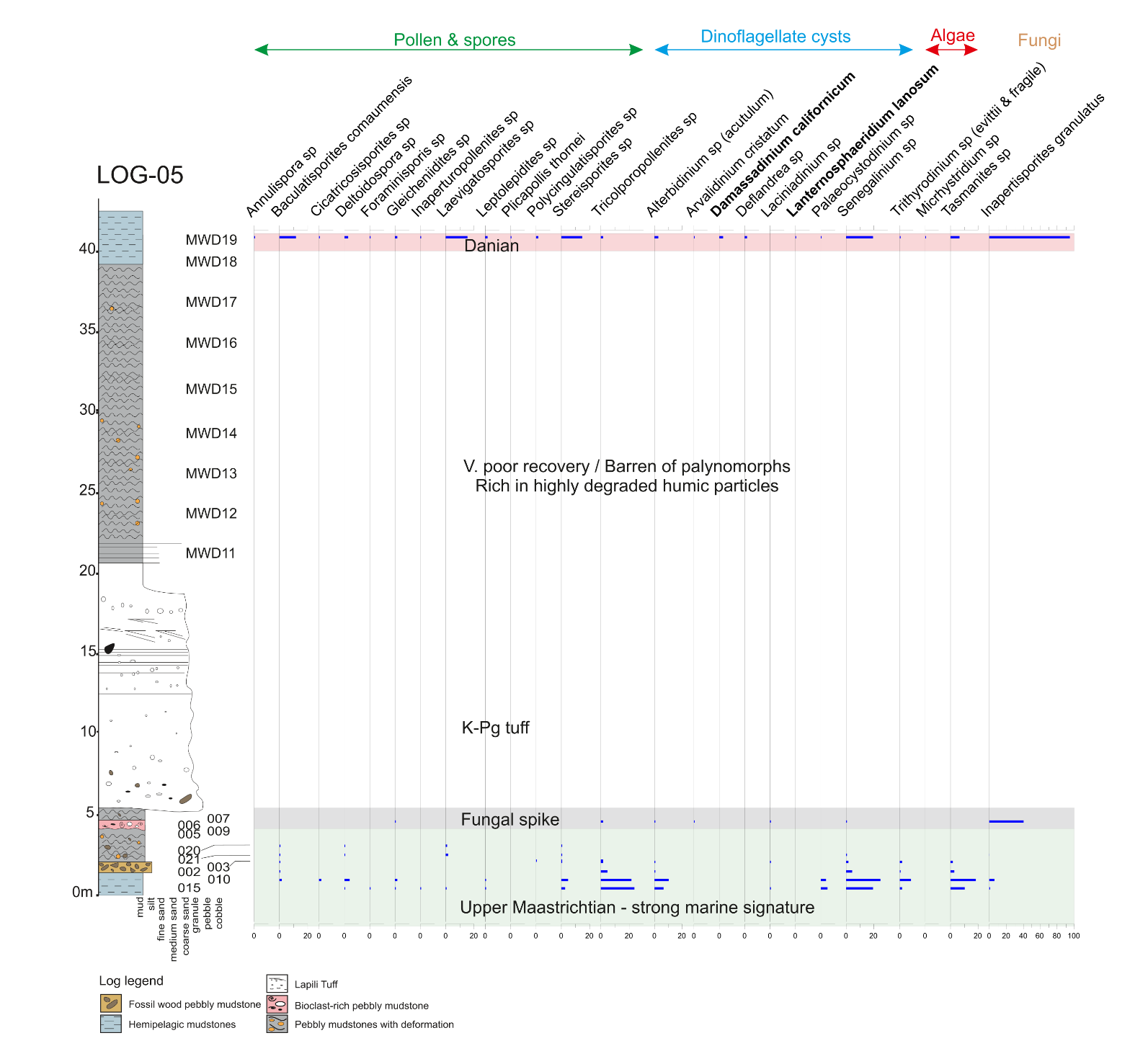


**Figure S2. Absolute counts of palynomorphs from the study interval.**

**Table S2. Palynomorph counts for samples from LOG-05.**

| **Sample name** | **Angiosperms** | **Bryophytes** | **Gymnosperms** | **Lycopsida** | **Pteridophytes** | **Miospore species** | **Dinocysts** | **Dinocyst species** | **Acritarchs** | **Acritarch sp.** | **Fungal spores** | **Total No species** |
| --- | --- | --- | --- | --- | --- | --- | --- | --- | --- | --- | --- | --- |
| MWD19 | 6 | 39 | 2 | 5 | 70 | 13 | 66 | 8 | 17 | 2 | 197 | 24 |
| MWD18 | 0 | 0 | 0 | 0 | 0 | 0 | 0 | 0 | 0 | 0 | 0 | 0 |
| MWD17 | 0 | 0 | 0 | 0 | 0 | 0 | 0 | 0 | 0 | 0 | 0 | 0 |
| MWD16 | 0 | 0 | 0 | 0 | 0 | 0 | 0 | 0 | 0 | 0 | 0 | 0 |
| MWD15 | 0 | 0 | 0 | 0 | 0 | 0 | 0 | 0 | 0 | 0 | 0 | 0 |
| MWD14 | 0 | 0 | 0 | 0 | 0 | 0 | 0 | 0 | 0 | 0 | 0 | 0 |
| MWD13 | 0 | 0 | 0 | 0 | 0 | 0 | 0 | 0 | 0 | 0 | 0 | 0 |
| MWD12 | 0 | 0 | 0 | 0 | 0 | 0 | 0 | 0 | 0 | 0 | 0 | 0 |
| LOG-05-07 | 16 | 0 | 0 | 0 | 8 | 2 | 33 | 4 | 0 | 0 | 342 | 7 |
| LOG-05-06 | 36 | 37 | 36 | 0 | 145 | 6 | 109 | 2 | 0 | 0 | 36 | 9 |
| LOG-05-20 | 266 | 133 | 0 | 0 | 0 | 5 | 0 | 0 | 0 | 0 | 0 | 0 |
| LOG-05-21 | 89 | 22 | 0 | 0 | 44 | 6 | 200 | 7 | 44 | 1 | 0 | 14 |
| LOG-05-03 | 0 | 0 | 0 | 0 | 0 | 0 | 0 | 0 | 0 | 0 | 0 | 0 |
| LOG-05-02 | 67 | 0 | 0 | 0 | 13 | 2 | 237 | 8 | 80 | 2 | 0 | 12 |
| LOG-05-01 | 16 | 0 | 0 | 0 | 24 | 5 | 194 | 7 | 100 | 2 | 0 | 30 |
| LOG-05-12 | 70 | 10 | 31 | 6 | 31 | 22 | 176 | 18 | 60 | 3 | 14 | 44 |
| LOG-05-18 | 96 | 12 | 14 | 3 | 12 | 15 | 200 | 21 | 58 | 5 | 6 | 42 |

**Table S3. Foraminifera specimens counted and identified on samples from LOG-05.**

|  | | | **FAMILY/SUPERFAMILY** | | | | | | | | | | | | | | | | | | | | |
| --- | --- | --- | --- | --- | --- | --- | --- | --- | --- | --- | --- | --- | --- | --- | --- | --- | --- | --- | --- | --- | --- | --- | --- |
|  |  |  | Ammodiscidae | Buliminellidae | Cibicidae | Gavelinellidae | | Haplophragmoididae | Hormosinellidae | Miliolidae | | | Nodosariidae | | | | | | Recurvoidinae | Reophacidae | Rhabdamminidae | Spiroplectamminidae | Verneuilinidae |
|  | | | **SPECIES** | | | | | | | | | | | | | | | | | | | | |
| Sample | Sediment (g) | Total | Ammodiscus californica | Buliminella obtusa | Cibicides sp | Gavelinella sp. | Gavelinella pertusa | Haplophragmoides suborbicularis | Hormosinelloides guttifer | Miliammina fusca | Silicosigmoilina californica | Quinqueloculina sp. | Dentalina sp. | Reussoolina apiculata | Lagena globosa | Favolagena supracretacea | Nodosaria filiformis | Lagena sp. | Cribrostomoides trinitatensis | Nodulina dentaliniformis | Bathysiphon sp. | Spiroplectammina spectabilis | Migros midwayensis |
| MWD-19 | 400 | 46 | 3 | 7 | 5 | 0 | 1 | 0 | 0 | 0 | 0 | 3 | 0 | 0 | 0 | 1 | 0 | 0 | 2 | 0 | 24 | 0 | 0 |
| MWD-18 | 400 | 200 | 9 | 10 | 9 | 5 | 8 | 0 | 0 | 9 | 3 | 10 | 0 | 3 | 0 | 34 | 0 | 55 | 8 | 0 | 37 | 0 | 0 |
| MWD-17 | 200 | 0 | 0 | 0 | 0 | 0 | 0 | 0 | 0 | 0 | 0 | 0 | 0 | 0 | 0 | 0 | 0 | 0 | 0 | 0 | 0 | 0 | 0 |
| MWD-16 | 200 | 0 | 0 | 0 | 0 | 0 | 0 | 0 | 0 | 0 | 0 | 0 | 0 | 0 | 0 | 0 | 0 | 0 | 0 | 0 | 0 | 0 | 0 |
| MWD-15 | 200 | 0 | 0 | 0 | 0 | 0 | 0 | 0 | 0 | 0 | 0 | 0 | 0 | 0 | 0 | 0 | 0 | 0 | 0 | 0 | 0 | 0 | 0 |
| MWD-14 | 200 | 3 | 0 | 0 | 0 | 0 | 0 | 0 | 0 | 0 | 0 | 0 | 0 | 0 | 0 | 0 | 0 | 0 | 0 | 0 | 0 | 3 | 0 |
| MWD-13 | 200 | 5 | 3 | 0 | 0 | 0 | 0 | 0 | 0 | 0 | 0 | 0 | 0 | 0 | 0 | 0 | 0 | 0 | 0 | 0 | 0 | 2 | 0 |
| MWD-12 | 200 | 1 | 0 | 0 | 0 | 0 | 0 | 0 | 0 | 1 | 0 | 0 | 0 | 0 | 0 | 0 | 0 | 0 | 0 | 0 | 0 | 0 | 0 |
| LOG-05-07 | 200 | 0 | 0 | 0 | 0 | 0 | 0 | 0 | 0 | 0 | 0 | 0 | 0 | 0 | 0 | 0 | 0 | 0 | 0 | 0 | 0 | 0 | 0 |
| LOG-05-21 | 400 | 200 | 17 | 4 | 12 | 0 | 0 | 0 | 0 | 5 | 36 | 6 | 22 | 8 | 3 | 36 | 1 | 34 | 5 | 0 | 0 | 7 | 4 |
| LOG-05-03 | 400 | 200 | 13 | 3 | 8 | 0 | 0 | 16 | 0 | 6 | 30 | 0 | 8 | 7 | 5 | 32 | 5 | 28 | 28 | 0 | 0 | 6 | 5 |
| LOG-05-02 | 200 | 0 | 0 | 0 | 0 | 0 | 0 | 0 | 0 | 0 | 0 | 0 | 0 | 0 | 0 | 0 | 0 | 0 | 0 | 0 | 0 | 0 | 0 |
| LOG-05-01 | 200 | 0 | 0 | 0 | 0 | 0 | 0 | 0 | 0 | 0 | 0 | 0 | 0 | 0 | 0 | 0 | 0 | 0 | 0 | 0 | 0 | 0 | 0 |
| LOG-05-12 | 400 | 200 | 10 | 4 | 25 | 13 | 0 | 25 | 14 | 7 | 11 | 3 | 4 | 22 | 0 | 16 | 2 | 13 | 17 | 13 | 1 | 0 | 0 |
| LOG-05-18 | 400 | 200 | 9 | 2 | 24 | 19 | 0 | 32 | 12 | 8 | 13 | 4 | 4 | 19 | 0 | 11 | 2 | 14 | 14 | 10 | 3 | 0 | 0 |

**Table S4: LOG-05-02 Paleocharcoal Data (3 s.f.)** ^[S1]^

| **Raman Parameter (FWHMRa) Results** | | |
| --- | --- | --- |
| FWHMRa | Minimum* | 1.54 |
|  | Maximum* | 2.54 |
|  | Median | 2.03 |
|  | Mean | 2.03 |
|  | Standard Deviation | 0.204 |
|  | Standard Error | 0.0292 |
| **FWHMRa Geothermometry Results** | | |
| Charring Temperature (°C) | Minimum | 395 |
|  | Maximum | 1022 |
|  | Median | 716 |
|  | Mean | 716 |
|  | Standard Deviation | 128 |
|  | Standard Error | 18.2 |

*Note maximum and minimum FWHMRa values do not correspond to maximum and minimum derived temperatures, given the inverse relationship between temperature and FWHMRa

A median temperature of 716°C is consistent with moderate-high intensity surface and/or crown fire conditions, whilst a maximum temperature of 1022°C indicates an origin within very intense fire. A minimum value of 395°C concurs with the lower temperatures of charcoal presentation in spectroscopic analysis. Sample spectrum 14 displayed very broad D and G bands, and intense fluorescence that rendered deconvolution inapplicable. Therefore, a temperature below 300°C can be broadly attributed to this spectrum, though it is not possible to discern more specific temperature estimates.

Visual assessment of spectra acquired present a range of notable characteristics that justify the Raman data collected, and temperatures calculated. Within these spectra, D(1) (main D-band, typically located at 1350 cm-1, that has been fit during spectral deconvolution) and G bands are typically clear and distinct, with the exception of spectrum A14, in which the bands are too highly obscured by fluorescence and signal ‘noise’. In those spectra that are not restricted by interference – though presentation is considerably variable – a range of additional band functions are apparent.

These functions present as distortions or shoulders on the main D(1) and G bands, and include bands D3, D4, D5 and D6. They represent additional features of the microstructure, though their study in charcoals is limited. Function D3, within these spectra, is typically seen as a distortion on the left shoulder of the G band, reducing gaussian fit overall. D6 is found on the right flank of the D(1) band, whilst D4 and D5 are typically amalgamated with D(1) to form a broad distorted shoulder. This broad shoulder in the D-band is lost at the highest temperatures. The band function D2, typically observed in thermal maturities seen beyond those experienced by charcoal, may in one instance be visible as a small distortion on the right shoulder of the G-band. Confidence in this observation is, however, limited by the presentation of fluorescence and noise in this spectrum.

Fig. S3 shows these features. The angle of the spectrum, caused by fluorescence, can been seen to reduce with increasing temperature. This is consistent with the association of fluorescence with low maturity material ‘contaminants’. As temperature of formation increases, D and G band widths reduce, particularly as the D band loses the broad left shoulder associated with D4 and D5 functions. The coherent change in width supports our application of FWHMRa. These spectra continue to highlight the inapplicability of intensity ratios in Raman-charcoal geothermometry, given the non-linear change in D-band height. The contrasting direction of shift in both D and G bands with increasing temperature supports established band separation relationships.


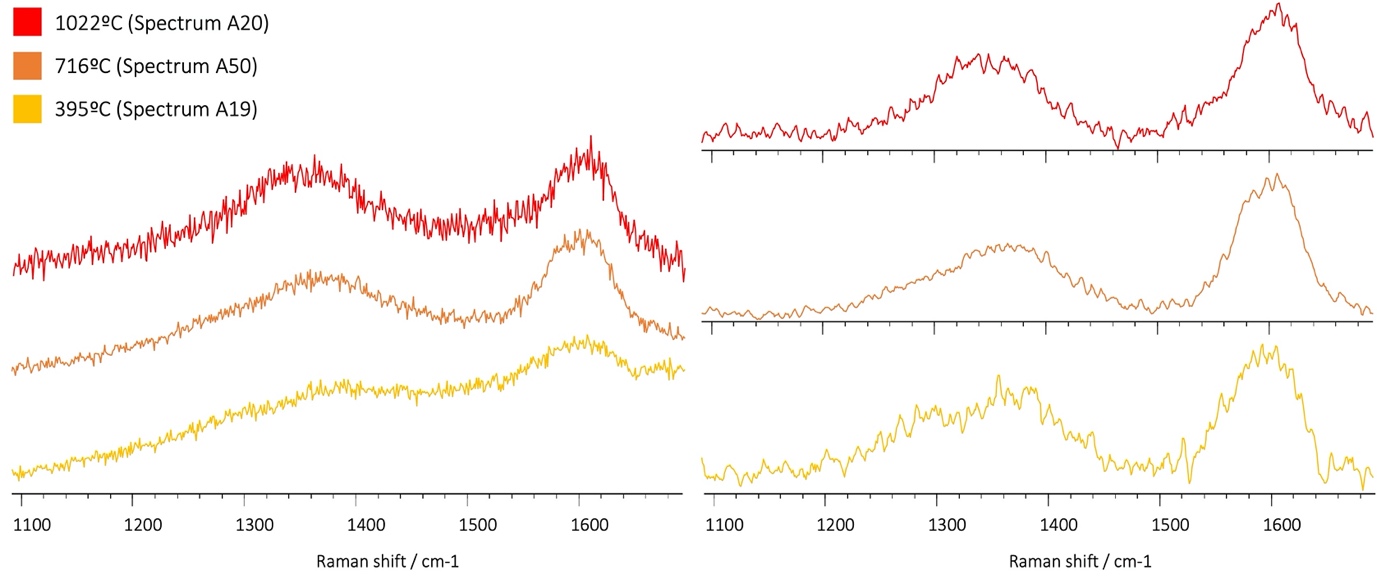


**Figure S3: raw (left) and deconvolved (right) spectra associated with highest, median, and lowest calculated temperatures.**

Analysis of this geothermometric data via histogram and boxplot presentation (Figs. S4, S5) highlight an apparent normality to this dataset. Outlying values, coinciding with maximum and minimum temperatures, occur at 395°C and 1022°C. The occurrence of normality in this dataset is further supported by ‘informal’ measures, including a standard deviation <50% mean value, and identical median and mean values. Statistical confirmation via the Shapiro-Wilk test (Table S5) indicates no significance, in which case the null hypothesis of assumed normality cannot be rejected. It is worth noting, however, that in this case the population (n = 49) falls below the standard minimum (50) for Shapiro-Wilk testing. Analysis of kurtosis and skew suggest a symmetrical dataset, with mild skew toward higher temperature values, respectively.

Normal distribution and unimodality exhibited by these data may suggest a single ‘population’ of paleocharcoals, derived from a single fire event. This conclusion, however, is most likely restricted by the small population under analysis (n = 49), and limited understanding of broad temperature presentation within natural fire systems.

**Table S5: Shapiro-Wilk test**

|  | Shapiro-Wilk Test | | | Kurtosis | Skewness |
| --- | --- | --- | --- | --- | --- |
|  | *n* | *W* | *p* |  |  |
| Log_05_02 Paleocharcoal | 49 | 0.984 | Not Significant | 0.281 | - 0.148 |


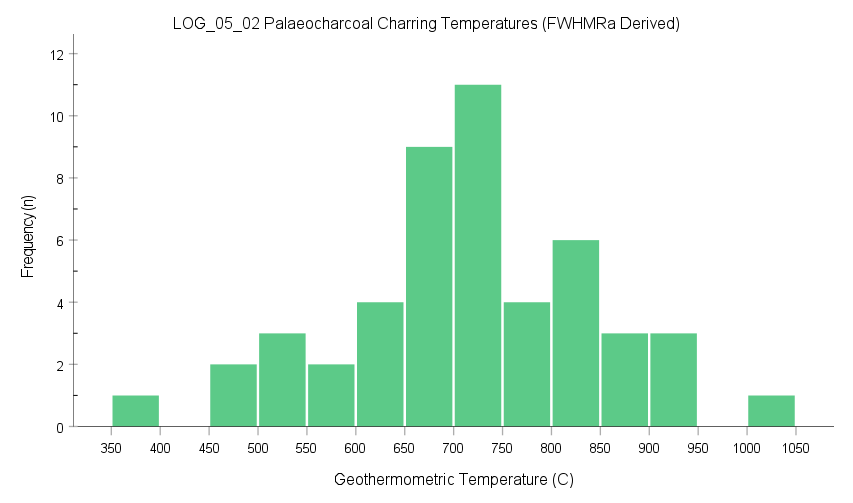


**Figure S4: Histogram of paleocharcoal charring temperatures (FWHMRa derived).**


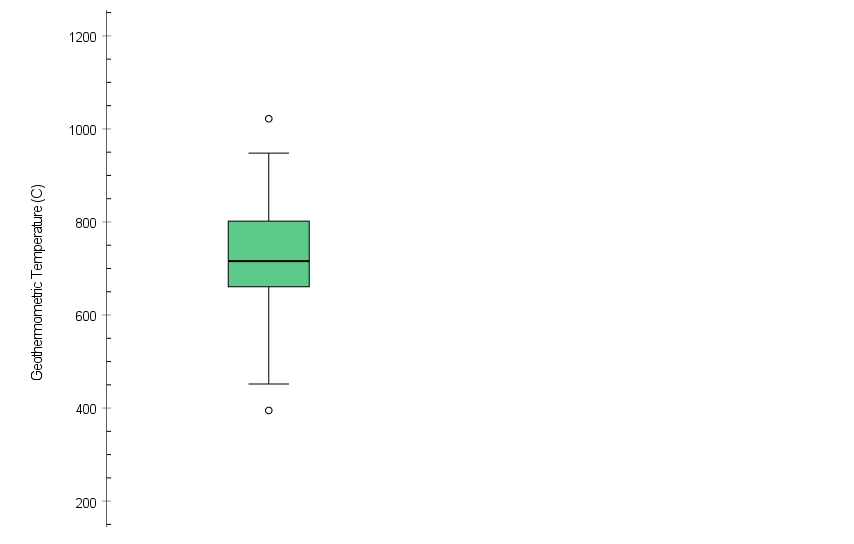


**Figure S5: Boxplot of paleocharcoal charring temperatures (FWHMRa derived).**

S1. Theurer, T., Naszarkowski, N., Muirhead, D., Jolley, D. and Mauquoy, D., 2022. Assessing Modern Calluna Heathland Fire Temperatures using Raman Spectroscopy: Implications for Past Regimes and Geothermometry. *Frontiers in Earth Science*.

**Table S6. Zircon grains analyzed from sample SF-30. Spots in green are from detrital grains, and in blue from an outlier; all 4 were excluded from the age calculation.**

|  | | | | | **204 corrected** | | | | | | | | | | |
| --- | --- | --- | --- | --- | --- | --- | --- | --- | --- | --- | --- | --- | --- | --- | --- |
| **Spot Name** | **% comm 206** | **ppm U** | **ppm Th** | **232Th /238U** | **238U/ 206Pb** | **% err** | **207Pb /206Pb** | **% err** | **207Pb /235U** | **% err** | **206Pb /238U** | **% err** | **err corr** | **206Pb**  **/238U Age** | **1s err** |
| SF30-1.1 | 0.58 | 281 | 142 | 0.52 | 91.162 | 2.3 | 0.04378 | 4.7 | 0.06622 | 5.3 | 0.01097 | 2.3 | 0.43 | **70.3** | 1.6 |
| SF30-2.1 | 0.09 | 166 | 93 | 0.58 | 93.785 | 2.4 | 0.04734 | 3.5 | 0.06961 | 4.3 | 0.01066 | 2.4 | 0.56 | **68.4** | 1.6 |
| SF30-3.1 | 0.67 | 63 | 22 | 0.36 | 94.854 | 3.6 | 0.04557 | 19.1 | 0.06624 | 19.4 | 0.01054 | 3.6 | 0.19 | **67.6** | 2.4 |
| SF30-4.1 | 1.36 | 132 | 93 | 0.72 | 96.316 | 2.5 | 0.04071 | 10.5 | 0.05827 | 10.7 | 0.01038 | 2.5 | 0.23 | **66.6** | 1.6 |
| SF30-5.1 | 0.24 | 352 | 175 | 0.51 | 97.105 | 2.2 | 0.04836 | 3.5 | 0.06868 | 4.1 | 0.01030 | 2.2 | 0.54 | **66.0** | 1.5 |
| SF30-6.1 | 0.18 | 688 | 484 | 0.73 | 95.225 | 2.2 | 0.04793 | 3.3 | 0.06940 | 3.9 | 0.01050 | 2.2 | 0.55 | **67.3** | 1.4 |
| SF30-7.1 | 0.14 | 494 | 392 | 0.82 | 96.295 | 2.2 | 0.04866 | 2.1 | 0.06968 | 3.0 | 0.01038 | 2.2 | 0.72 | **66.6** | 1.4 |
| SF30-8.1 | 0.49 | 400 | 213 | 0.55 | 97.745 | 2.2 | 0.04683 | 5.6 | 0.06606 | 6.0 | 0.01023 | 2.2 | 0.37 | **65.6** | 1.5 |
| SF30-9.1 | 0.42 | 381 | 216 | 0.59 | 94.950 | 2.3 | 0.05091 | 8.7 | 0.07393 | 9.0 | 0.01053 | 2.3 | 0.25 | **67.5** | 1.5 |
| SF30-10.1 | 0.05 | 151 | 75 | 0.51 | 61.418 | 2.4 | 0.05010 | 5.3 | 0.11248 | 5.8 | 0.01628 | 2.4 | 0.41 | **104.1** | 2.5 |
| SF30-11.1 | 5.01 | 77 | 41 | 0.55 | 99.955 | 2.7 | 0.04623 | 16.1 | 0.06372 | 16.4 | 0.01000 | 2.7 | 0.17 | **64.2** | 1.7 |
| SF30-12.1 | 0.22 | 517 | 333 | 0.67 | 73.535 | 2.2 | 0.04794 | 4.1 | 0.08989 | 4.7 | 0.01360 | 2.2 | 0.46 | **87.1** | 1.9 |
| SF30-13.1 | 0.00 | 315 | 235 | 0.77 | 95.104 | 2.3 | 0.04777 | 2.8 | 0.06925 | 3.6 | 0.01051 | 2.3 | 0.63 | **67.4** | 1.5 |
| SF30-14.1 | 0.55 | 576 | 424 | 0.76 | 94.944 | 2.2 | 0.04776 | 7.0 | 0.06936 | 7.3 | 0.01053 | 2.2 | 0.30 | **67.5** | 1.5 |
| SF30-15.1 | 0.04 | 390 | 69 | 0.18 | 72.084 | 2.2 | 0.04903 | 2.7 | 0.09378 | 3.5 | 0.01387 | 2.2 | 0.63 | **88.8** | 1.9 |
| SF30-16.1 | 0.02 | 223 | 187 | 0.86 | 97.345 | 2.3 | 0.04823 | 3.3 | 0.06831 | 4.0 | 0.01027 | 2.3 | 0.58 | **65.9** | 1.5 |
| SF30-17.1 | 1.35 | 146 | 44 | 0.31 | 97.586 | 2.5 | 0.04446 | 8.7 | 0.06281 | 9.1 | 0.01025 | 2.5 | 0.27 | **65.7** | 1.6 |
| SF30-18.1 | 0.69 | 293 | 226 | 0.80 | 93.687 | 2.3 | 0.04551 | 5.5 | 0.06698 | 6.0 | 0.01067 | 2.3 | 0.38 | **68.4** | 1.6 |
| SF30-19.1 | 0.31 | 211 | 85 | 0.42 | 97.764 | 2.4 | 0.04638 | 7.5 | 0.06540 | 7.9 | 0.01023 | 2.4 | 0.30 | **65.6** | 1.5 |
| SF30-20.1 | 0.55 | 248 | 135 | 0.56 | 98.121 | 2.3 | 0.04710 | 4.3 | 0.06617 | 4.9 | 0.01019 | 2.3 | 0.47 | **65.4** | 1.5 |
| SF30-21.1 | 1.77 | 166 | 72 | 0.45 | 96.404 | 2.4 | 0.04689 | 7.8 | 0.06705 | 8.2 | 0.01037 | 2.4 | 0.29 | **66.5** | 1.6 |
| SF30-22.1 | 0.42 | 216 | 123 | 0.59 | 98.334 | 2.4 | 0.04727 | 9.2 | 0.06627 | 9.5 | 0.01017 | 2.4 | 0.25 | **65.2** | 1.5 |
| SF30-23.1 | 0.00 | 184 | 74 | 0.41 | 98.107 | 2.7 | 0.04727 | 3.6 | 0.06643 | 4.5 | 0.01019 | 2.7 | 0.61 | **65.4** | 1.8 |
| SF30-24.1 | 0.00 | 303 | 138 | 0.47 | 97.514 | 2.3 | 0.04670 | 3.1 | 0.06603 | 3.8 | 0.01025 | 2.3 | 0.59 | **65.8** | 1.5 |
| SF30-25.1 | 0.80 | 194 | 99 | 0.53 | 101.350 | 2.4 | 0.04633 | 7.5 | 0.06303 | 7.9 | 0.00987 | 2.4 | 0.30 | **63.3** | 1.5 |
| SF30-26.1 | 0.00 | 185 | 91 | 0.51 | 96.515 | 2.4 | 0.05277 | 3.3 | 0.07538 | 4.1 | 0.01036 | 2.4 | 0.58 | **66.4** | 1.6 |
| SF30-27.1 | 0.45 | 131 | 43 | 0.34 | 100.288 | 3.0 | 0.04756 | 24.9 | 0.06538 | 25.1 | 0.00997 | 3.0 | 0.12 | **64.0** | 1.9 |
| SF30-28.1 | 0.92 | 180 | 61 | 0.35 | 99.831 | 2.8 | 0.04349 | 8.7 | 0.06006 | 9.1 | 0.01002 | 2.8 | 0.31 | **64.2** | 1.8 |


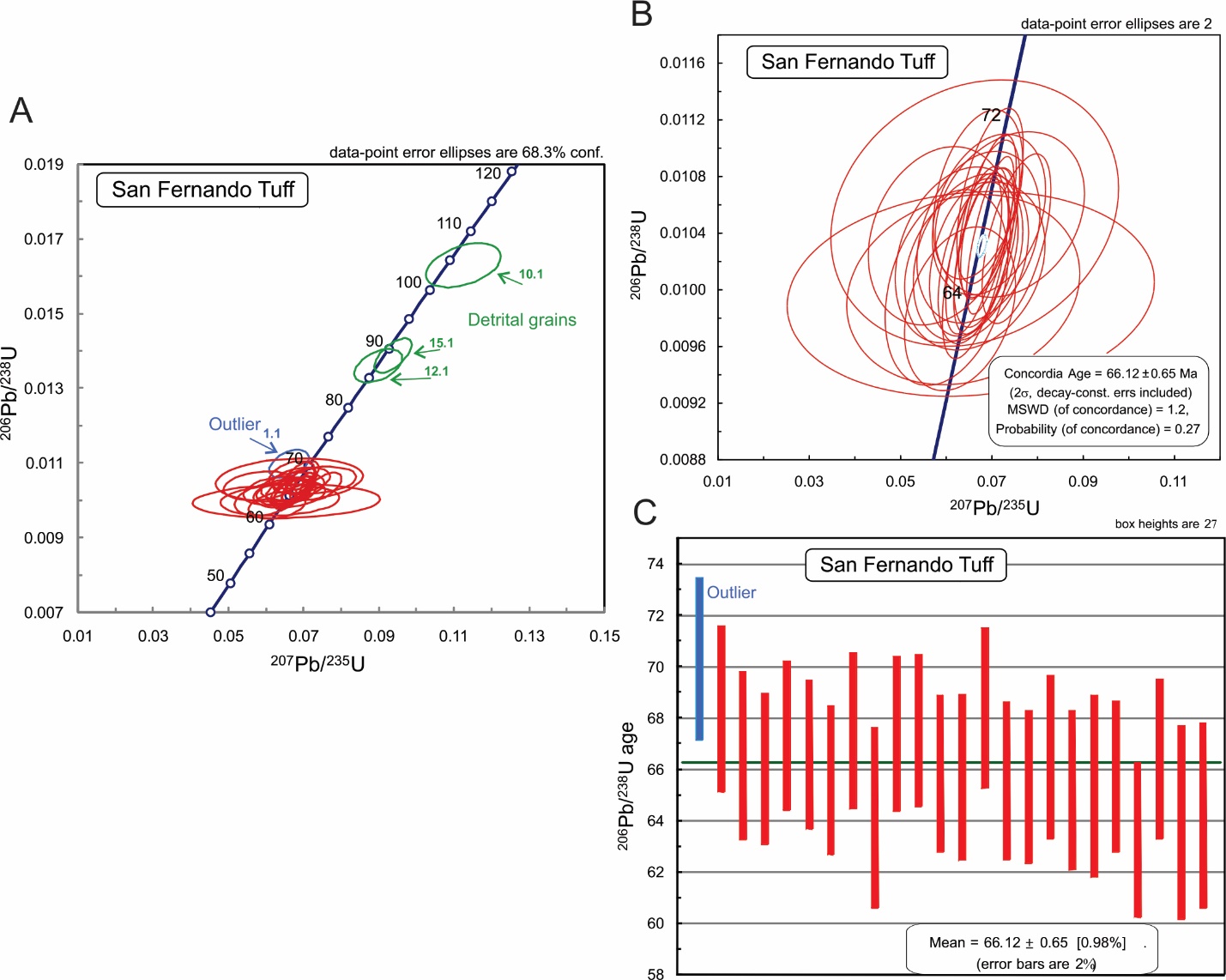


**Figure S6. Diagrams showing the results of analyzed zircon spots. A) Concordia diagram with the distribution of all analyzed spots, including detrital or inherited older grains in green and an outlier spot in blue; B) Concordia diagram showing Concordia age (2 sigma error) obtained after excluding older grains and the outlier, and C) ^206^Pb/^238^U weighted mean age (2 sigma error) excluding older grains and showing the outlier (not included in the age calculation).**

**Table S7. Results of iridium analysis for selected samples of LOG-05.**

| **Analyte Symbol** | **LOI (%)** | **Total (%)** | **Ir (ppb)** | **Mass (g)** |
| --- | --- | --- | --- | --- |
| **Detection limit** |  | **0,01** | **0,1** |  |
| **Analysis method** | **FUS-ICP** | **FUS-ICP** | **NI-FINA** | **NI-FINA** |
| MWD-18 | 8.96 | 99.81 | 1.2 | 15 |
| LOG-05-07 | 8.94 | 99.13 | 0.5 | 15 |
| LOG-05-21 | 10.18 | 99.07 | 0.3 | 15 |
